# Evaluating the Development, Reliability, and Validation of the Tele-Primary Care Oral Health Clinical Information System Questionnaire: Cross-Sectional Questionnaire Study

**DOI:** 10.2196/53630

**Published:** 2025-01-29

**Authors:** Rosnah Sutan, Shahida Ismail, Roszita Ibrahim

**Affiliations:** 1 Department of Public Health Medicine Faculty of Medicine Universiti Kebangsaan Malaysia Cheras Kuala Lumpur Malaysia

**Keywords:** telehealth, electronic health, eHealth, public health information system, psychometric analysis

## Abstract

**Background:**

Evaluating digital health service delivery in primary health care requires a validated questionnaire to comprehensively assess users’ ability to implement tasks customized to the program’s needs.

**Objective:**

This study aimed to develop, test the reliability of, and validate the Tele-Primary Care Oral Health Clinical Information System (TPC-OHCIS) questionnaire for evaluating the implementation of maternal and child digital health information systems.

**Methods:**

A cross-sectional study was conducted in 2 phases. The first phase focused on content item development and was validated by a group of 10 experts using the content validity index. The second phase was to assess its psychometric testing for reliability and validity.

**Results:**

A structured questionnaire of 65 items was constructed to assess the TPC-OHCIS delivery for primary health care use based on literature and has been validated by 10 experts, and 319 respondents answered the 65-item TPC-OHCIS questionnaire, with mean item scores ranging from 1.99 (SD 0.67) to 2.85 (SD 1.019). The content validity, reliability, and face validity showed a scale-level content validity index of 0.90, scale-level content validation ratio of 0.90, and item-level face validity index of 0.76, respectively. The internal reliability was calculated as a Cronbach α value of 0.90, with an intraclass correlation coefficient of 0.91. Scales were determined by the scree plot with eigenvalues >1, and 13 subscales were identified based on principal component analysis. The Kaiser-Meyer-Olkin value was 0.90 (*P*<.049). The total variance explained was 76.07%, and factor loading scores for all variables were >0.7. The Bartlett test of sphericity, determining construct validity, was found to be significant (*P*<.049).

**Conclusions:**

The TPC-OHCIS questionnaire is valid to be used at the primary health care level to evaluate the TPC-OHCIS implementation. It can assess health care workers’ work performance and job acceptance and improve the quality of care.

## Introduction

### Background

Digital health is mostly used interchangeably with eHealth, telehealth, or mobile health in the literature [1,2]. It requires integrated and interdisciplinary sector involvement to use knowledge information and communication technology in health (eg, medicine, public health, pharmaceutical, dentistry, and health management) [1-8]. It enables the national health system to ensure population access to health services and the ability to monitor and evaluate health system delivery performance [1,2,9]. Electronic health records are created from digital health systems for case management monitoring and can be shared across health care settings [9]. The data sharing platform allows access to integration interfaces that include electronic medical records, appointments, electronic prescriptions, e-commerce, public health surveillance, system monitoring such as vaccination, environmental health, institutional health management, and an online platform for teaching and learning among health care workers (HCWs) [10-16].

### Malaysian Digital Health

Malaysia began integrating digital health into its health care system in 1998 [4,5]. The Telemedicine Act 1997 (Act 564) was enacted to regulate telemedicine practices in the country [4,5,17-25]. Later, the telemedicine blueprint was created to outline the government’s vision for digital health implementation and align it with the 7 National Multimedia Super Corridor flagship applications [19-22]. The initiative was referred to as telemedicine and later restated as telehealth. The telehealth system serves as a platform for digital health services in Malaysia [20]. The National Telehealth Policy was launched to support the Vision 2020 agenda, focusing on four key components: (1) lifetime health plan, (2) mass customized health information and education, (3) continuous medical education, and (4) teleconsultation application [1]. The policy was formulated during the Eighth Malaysia Plan and managed by the National Health Informatics Centre Division [4,17,19-22]. The lifetime health plan covers health services from womb to tomb [19]. The telehealth system also includes the clinical support system and health information management and support services, and it encompasses the hospital information system and the Tele-Primary Care Oral Health Clinical Information System (TPC-OHCIS) [18-24]. The TPC-OHCIS is a comprehensive electronic medical record system for primary health care [22-24]. It was initially developed for outpatient services at primary health care clinics (PHCs), and later, it incorporated maternal and child health (MCH), oral health, and other life stage health services to ensure continuity of care [21,22]. The development of TPC-OHCIS was a collaborative effort between the Ministry of Science, Technology, and Innovation; the Ministry of Health, Malaysia; and the Malaysian Institute of Microelectronic Systems, Berhad [21-24]. The system was first tested in the PHCs of Seremban District, Negeri Sembilan, and later expanded to 34 PHCs across 3 states (ie, Perlis, Sarawak, and Selangor) [17,21]. To date, the TPC-OHCIS has been implemented in 108 PHCs across 7 additional states [22,25]. The TPC-OHCIS is a web-based platform that allows HCWs to enter data during clinic services or home visits, automatically updating when connected to the internet [23,24].

### Role of Digital Health in Service Quality Performance

The recent COVID-19 pandemic revealed a significant public health issue in health care system delivery to provide comprehensive quality care [2,26-28]. Malaysia has experienced various health service delivery disruptions at PHCs during the critical phase of the COVID-19 pandemic [29]. MCH services include a wide range of services covering school health programs that experienced substantial disruptions due to closing and movement control orders, thus preventing mothers and children from receiving adequate health care services [22]. During the COVID-19 pandemic, many countries have improved health care delivery through digital technology, enhanced resource coordination, and facilitated universal health coverage [29,30].

The HCWs at the PHCs worked on the frontlines, assessing risks, monitoring care treatment, and promoting health empowerment in the community [17]. Most of the administration work related to patient care is recorded manually. Even with the implementation of TPC-OHCIS, many facilities still need to record data manually and enter it into the system because of internet instability at some PHCs [22]. At present, there is no specific policy published by the Ministry of Health to completely replace manual recording of patient information monitoring with digital health. The HCWs monitor patients during home visits and conduct outreach activities to cover areas inaccessible to health facilities [17,22]. Therefore, it is important to continue patient using manual data recording when services are provided offline. The TPC-OHCIS is an electronic medical record system used as a daily operating system and in “real time” at PHCs [22-24]. However, the TPC-OHCIS implemented in selected facilities in Malaysian PHCs is used mostly to record data and monitor patient care only [22-24].

### Specific Study Measurement Tool

The conceptualization of this study diverges from earlier studies primarily in its integration of advanced technological modalities and emphasis on patient-centered care delivery, unlike traditional telehealth frameworks, which often focus on providing remote consultations or basic medical services [1,3,18,25]. The TPC-OHCIS incorporates elements of comprehensive primary care delivery, leveraging telehealth technologies to facilitate longitudinal patient-provider relationships, care coordination, and proactive health management. Moreover, this study’s conceptualization places a heightened emphasis on the integration of patient health data, wearable devices, and digital health platforms to enhance care delivery and patient engagement. The TPC-OHCIS can remotely monitor patient health metrics, deliver personalized interventions, and empower individuals to take an active role in their health management [24]. The importance of interdisciplinary collaboration and team-based care is crucial in the implementation of the TPC-OHCIS at PHCs. Hence, it is important to assess the implementation of TPC-OHCIS by focusing on technology, organization, environment, or human resource components, as suggested in the literature [3-5,9]. Earlier studies were conducted to evaluate the effectiveness of the TPC-OHCIS in improving health care quality; however, there is limited evidence on assessing HCWs’ perceived usefulness and ease of use [9,18,25]. A comprehensive study of HCWs’ perspectives is important to provide a shred of extensive knowledge and evidence on the TPC-OHCIS implementation in PHCs.

Therefore, this study aimed at developing, testing reliability, and validating the TPC-OHCIS questionnaire, which was designed as a survey instrument to collect data on the implementation of the TPC-OHCIS at the PHC setting among HCWs related to various aspects of primary care services, including MCH services. It aims to ensure that the TPC-OHCIS questionnaire is a robust and effective tool for assessing the implementation of MCH services, which are the core services for PHCs. The research question formulated was as follows: “Is a customized questionnaire on digital health information system (TPC-OHCIS) able to assess the HCWs’ perception of its implementation process in the delivery of MCH services at the primary care level?” A thorough design of the TPC-OHCIS questionnaire validation helps increase the questionnaire’s relevance and usefulness for decision-making purposes, as it is designed to facilitate monitoring and evaluating the TPC-OHCIS operability among the HCWs working at PHCs. To the best of our knowledge, there is no valid questionnaire available to measure the implementation evaluation of any digital health information system for the primary care level that focuses on MCH services, which is a priority service component of PHC and which was monitored regularly for sustainable development goals performance achievement [17].

## Methods

### Study Design

This study involved several steps for questionnaire development (phase 1), reliability, and validation (phase 2).

### Phase 1: Questionnaire Development

The questionnaire is adapted from literature and document review [4,5,9,17-25,31-36]. The TPC-OHCIS questionnaire was developed based on various theoretical models, which may address multiple aspects of remote primary care and patient information systems. The questionnaire was created based on a combination of the technology-organization-environment (TOE) theory [31], the technology acceptance model (TAM) theory [32,33], the human organization technology-fit (HOT-fit) model [34,35], and the diffusion of innovation (DOI) theory [36]. There was a 65-item questionnaire with a 4-point Likert scale (1=highly disagree, 2=disagree, 3=agree, and 4=highly agree) developed based on the aforementioned theories [31-36]. The score scale is created according to the Likert scale that indicates the following: 1=strongly agree, 2=agree, 3=disagree, and 4=strongly disagree. In this study, the researcher did not put a neutral on a scale of 3 to avoid respondent bias [37]. The 4-point Likert scale does not impact the reliability and validity of the questionnaire [2,37].

The development of the questionnaire items was partly adapted from various questionnaires available from previous studies conducted in Malaysia, based on selected theoretical models, using a 4-point scale questionnaire: (1) technology, (2) organization, (3) environment, and (4) human [31-36] (Multimedia Appendix 1 [4,5,9,17-25,31-36]). Therefore, we classified the 65-item TPC-OHCIS questionnaire into 4 scales (Multimedia Appendix 2), described below.

- Domain A, technology: this comprised 17 items and four subscales that include (1) relative advantage (items 1-5), (2) compatibility (items 6-9), (3) complexity (items 10-13), and (4) security concern (items 14-17).
- Domain B, organization: this contained 18 items and four subscales that include (1) the presence of a specified liaison officer (BCHAMP: items 18-22), (2) infrastructure (BINFRA: items 23-26), (3) top management support (BTP: items 27-31), and (4) financial resources (BFIN: items 32-35).
- Domain C, environment: this focuses on vendor support (CVEN: items 36-39).
- Domain D, human: this contained 26 items and six subscales that include (1) staff competency in information systems (DPT: items 40-44), (2) knowledge of the TPC-OHCIS system (DEISK: items 45-50), (3) clinical information technology competency (DCIT: items 51-54), (4) perceived innovativeness of the IT officer (DCIO: items 55-57), (5) perceived ease use (DPEU: 58-61), and (6) perceived usefulness (DPU: items 62-65).

The 65-item TPC-OHCIS questionnaire was developed based on the requirement of the TPC-OHCIS implementation plan for the PHCs in Malaysia. A total of 15 items (item numbers 10, 11, 12, 13, 33, 40, 41, 42, 48, 49, 50, 53, 54, 58, and 39) were written as negative items. The Likert scale score of negative items was reversed for scoring analysis before data were entered into the SPSS software (version 26.0; IBM Corp).

### Phase 2: Questionnaire Validation

On the basis of the steps suggested by Boateng et al [38], the second phase involved scale development, which consists of pretesting questions, sampling and survey administration, item reduction, and extraction of latent factors. The scale evaluation requires tests of dimensionality, reliability, and validity. A cross-sectional study was conducted to assess the psychometric properties of the questionnaire.

### Study Sample

The sample for each step was calculated and listed in Table 1.

The sample-to-item ratio was decided based on the number of items in the questionnaire. The ratio per item was determined using 5 to 20 samples per item [39,40]. In this study, the survey included 65 items. Therefore, the total calculated sample size required was 325 respondents.

**Table 1:** Calculated sample size for each validation step.

Steps and aims	Sample size required, n	Reference
Pilot study and face validity: to test the adequacy of instrumentation in which the outcome is in the form of a scale	40	[3]
Test-retest reliability: to test the degree of consistency exhibited when a measurement is repeated under identical conditions	12	[4]
Field survey: to test the psychometric analysis of the questionnaire based on tests of dimensionality, reliability, and validity	325	[5]

### Content Validity Index

**Figure 1:**
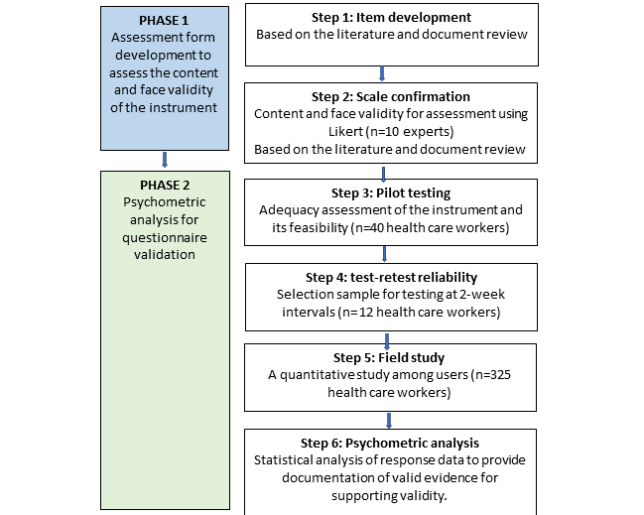
Flow process for questionnaire validation.

The flow process of questionnaire validation was conducted based on Figure 1.

On the basis of Figure 1, a total of 6 steps were taken to perform content validity for the questionnaire.

#### Step 1: Assessment Form Development for Content and Face Validity

In our literature review search [37,39-41], we listed items to determine the content and face validity. We used the clarity scale of 1 to 3 to indicate the relevance of each item (1=not clear, 2=revision needed, and 3=very clear). The essentiality of the questionnaire was identified using a 1 to 3 scale (1=not essential, 2=useful but not essential, and 3=essential). A similar form was used to assess face validity in evaluating the language, feasibility, readability, and style formatting consistency [37,41].

#### Step 2: Content and Face Validity (Expert Review)

Face validity was conducted among the experts who were involved in designing and using the system at the primary care level. Ten experts were invited to review the 65-item Tele-Primary Care Oral Health Clinical Information System (TPC-OHCIS) questionnaire. All experts have extensive experience and knowledge related to the TPC-OHCIS. Of the 10 experts, 3 (30%) were from the TPC-OHCIS division, 3 (30%) were public health researchers, 2 (20%) were from the maternal and child health unit services in the Selangor State Health Department, and finally, 2 (20%) were individuals who worked directly with the TPC-OHCIS system and also served as system liaison officers at health clinics. The adaptation of questionnaires from earlier studies [41-43] was checked for content suitability and appropriateness before forward translation was performed. Scale evaluation was performed by experts using a developed form. Owing to time constraints and lockdown because of the COVID-19 pandemic, a face-to-face method for collecting data from the experts was impossible. A Google form (Google LLC) link was created and sent via WhatsApp (Meta Platforms, Inc) to assess the face and content validity. The selected 10 experts were asked to evaluate the scale for content and face validation. All 10 experts’ comments were used to improve the questionnaire. Experts were encouraged to provide both verbal and written comments regarding the questionnaire items. Verbal comments were collected via phone call, and written comments were collected via the Google form.

#### Step 3: Pilot Study

A pilot study was conducted to assess the adequacy of the instrumentation, which measured outcomes to ensure that the instruments used were appropriate and effective for the intended purpose. Through the pilot study, face validity was evaluated to confirm that the 65-item TPC-OHCIS questionnaire developed was constructed accurately and comprehensively. We purposively invited health care workers (HCWs) who were involved in the initial pilot-tested version of the TPC-OHCIS in Seremban District, Negeri Sembilan [17]. The questionnaire was sent after a thorough face and content validity assessment by the experts. Correction and fine-tuning were conducted by the researchers based on their feedback.

#### Step 4: Test-Retest Reliability

We selected 12 HCWs who were familiar with the TPC-OHCIS involved in the pilot study mentioned above to test the degree of consistency exhibited when a measurement is repeated under identical conditions. The same questionnaire was sent to the same respondents 2 weeks after the first time the survey was conducted.

#### Step 5: Field Study

A cross-sectional study was performed to assess the item reduction and extraction factors among the HCWs of the TPC-OHCIS public health care clinics (PHCs) in selected states in Malaysia. The TPC-OHCIS is a system used in the PHCs. The PHC is headed by a family medicine specialist who supervises staff from various disciplines (eg, physicians, nurses, medical assistants, pharmacists, laboratory technicians, and record officers).

#### Step 6: Performing an Analysis for Content Validity, Face Validity, and Test-Retest Reliability

The item-level content validity index (I-CVI) and scale-level content validity index (S-CVI) were used. I-CVI is defined as the proportion of content experts giving items a relevance rating of 3 and 4. S-CVI was calculated by the proportion of understanding by experts (universal agreement [UA] of S-CVI), which gave ratings of 3 and 4, or by average scores given (average of S-CVI). The average of S-CVI is defined as the average of the I-CVI scores for all items on the scale or the average of proportion relevance judged by all experts. The UA of S-CVI is the proportion of items on the scale that achieve a relevance scale of 3 or 4 by all experts. UA score is given as 1 when the item achieved 100% agreement by all experts; otherwise, the UA score is given as 0 [39]. First, we calculated the experts in agreement. Second, a score of 1 was given to items that achieved 100% agreement by all experts, while a score of 0 was given to items that did not achieve 100% agreement. Third, experts in the agreement were divided by the total number of experts (calculated for I-CVI). The fourth step was to divide the results obtained in the third step by 65, which was the total number of items in the questionnaire (calculated for average of S-CVI). Finally, we divided the UA result from step 2 by 65 items (calculated for UA of S-CVI). The face validity index was used to evaluate the form of clarity and comprehensibility of language and instructions used in the questionnaire [41]. The respondents were requested to rate the comprehensibility of each item using a scale of 1 to 4 (1=not understandable, 2=somewhat understandable, 3=understandable, and 4=very understandable). The item-level face validity index was computed for each item by dichotomizing the 4-point scale, where items scoring either 1 or 2 were recorded as 0 and items scoring 3 and 4 were recorded as 1. The values later were added up according to each item, and the total values were divided by the total number of respondents. Test-retest reliability was used to define whether the questionnaire was answered by respondents due to chance. The internal reliability of questionnaires was assessed using the Cronbach α coefficient. The Pearson correlation and intraclass correlation coefficients of the scores of the 2 tests were calculated. The test-retest reliability is achieved when the value of intraclass correlations is 0.6 to 0.8 (good reliability), and values >0.8 indicate excellent reliability, which means the higher the correlation, the higher the test-retest reliability, with values close to 0 indicating low reliability [38].

### Forward-Backward Translation

The questionnaire was translated from English to the Malay language, then underwent content assessment, and its cross-cultural validity was evaluated by 3 experts representing Malaysia’s diverse ethnic backgrounds (Malay, Chinese, and Indian), proficient in both English and Malay. After the questionnaire had been reviewed, the forward-backward translation was sent to Proofreaders United company for translation and proofreading.

### Ethical Considerations

Ethics approval was granted by the National University of Malaysia (FF-2021-124) and the Ministry of Health Malaysia National Medical Research Registry (NMRR-21-599-58521, investigatory initiated research). Meanwhile, the researcher also needed to obtain verbal permission and a signed consent letter from the respondent as evidence of consent to participate in the study. Respondents were informed that their participation was voluntary and they would not receive any payment. All research information collected was treated as confidential. The data are displayed anonymously without the name, address, or any identity that describes the respondent when presented as the study output.

### Statistical Analysis

The internal reliability was assessed using Cronbach α. The validity index was calculated based on the content validity index (CVI). The Bartlett test of sphericity was used to determine the construct validity. The Kaiser-Meyer-Olkin measure and total variance explained (TVE) were evaluated. Factor analysis was performed using varimax rotation in principal component analysis (PCA) to verify construct validity (ie, discriminant and convergent validity). Items loading >0.60 were considered for further analysis [38].

## Results

### CVI Evaluation

Out of 10 experts evaluating the 65-item TPC-OHCIS questionnaire (TPC-OHCIS questionnaire in Multimedia Appendix 2), the scale-level CVI (S-CVI) average for item 1 of domain A, relative advantage, to item 65 of domain D, perceived usefulness, was 0.9. The average of S-CVI (based on item-level CVI) was 7.415, the average of S-CVI (based on proportion) was 0.742, and the universal agreement of S-CVI was 0.045. The universal agreement of S-CVI was calculated based on the proportion of items on the scale that obtained a relevance value of 3 or 4 from all the experts (Multimedia Appendix 3).

### Face Validation

The face validity index was assessed for clarity, language comprehensibility, and instructions used in the questionnaire. The calculated item face validity index was 0.785 and fulfilled the face validity criteria, which was >0.6 [41].

### Test-Retest Reliability

The 65-item survey’s internal reliability score for Cronbach α was 0.90 for the whole instrument and exceeded the suggested minimum value of Cronbach α=0.70 [38]. Pearson correlation coefficient was 0.90, and the intraclass correlation coefficient was 0.91.

### Descriptive Analysis

There were 319 respondents (Table 2) who answered the 65-item TPC-OHCIS questionnaire, with mean item scores ranging from 1.99 to 2.85 (Table 3). Our study found that a high mean score was achieved in all subscales except for the item 17 in domain A, security concerns (*how much can I trust the vendor)*. The subscale mean scores were lowest at 1.93 (SD 0.751) and 1.99 (SD 0.832) for item 63 in domain D, perceived usefulness (*TPC-OHCIS application improved the quality of my work, respectively).* The mean score for communication was the highest for item 38 in domain C, vendor, with a mean score of 3.45 (SD 0.819), and item 39 in domain C, vendor, with a mean score of 3.39 (SD 0.805).

**Table 2:** Respondent profile for field study (N=319).

Variables and category		Frequency, n (%)
**Occupation**		
	Family medicine specialist	5 (1.6)
	Medical officer	69 (21.6)
	Matron	2 (0.6)
	Head nurse	17 (5.3)
	Nurse	138 (43.3)
	Community nurse	88 (27.6)
**Sex**		
	Male	6 (1.9)
	Female	313 (98.1)
**Race**		
	Chinese	7 (2.2)
	Indian	21 (6.6)
	Malay	285 (89)
	Others	6 (1.9)

**Table 3:** Descriptive statistics for item mean score of the Tele-Primary Care Oral Health Clinical Information System (TPC-OHCIS) questionnaire.

Item question	Question statement	Scores, mean (SD)
ARA^a^ 1	Using the TPC-OHCIS application enables me to do my work quickly.	2.05 (0.865)
ARA 2	Using the TPC-OHCIS application improves the quality of my work.	1.99 (0.832)
ARA 3	Using the TPC-OHCIS application enhances my effectiveness on the job.	2.01 (0.864)
ARA 4	Using the TPC-OHCIS application increases my productivity.	2.04 (0.825)
ARA 5	Using the TPC-OHCIS application makes my job easier.	2.02 (0.795)
ACOM^b^ 6	TPC-OHCIS application can be easily accessed across multiple platforms (laboratory results, x-ray, and other related patient data).	2.53 (0.941)
ACOM 7	TPC-OHCIS user interfaces provide transparent access to all platforms (e-notification and VEKPRO^c^).	2.62 (0.931)
ACOM 8	Data received from other devices (tablet/laptop/ smartphone) outside health facilities in the TPC-OHCIS application can be easily merged into the database for analysis.	2.74 (0.932)
ACOM 9	Information is shared seamlessly across our organization regardless of location.	2.74 (0.839)
ACOMPLEX^d^ 10	I do not know enough about the TPC-OHCIS application to handle my job satisfactorily.	2.86 (0.870)
ACOMPLEX 11	I need a long time to understand and get familiar with the TPC-OHCIS application.	2.82 (0.961)
ACOMPLEX 12	I do not find enough time to study and upgrade my technology skills before using the TPC-OHCIS system.	2.71 (0.870)
ACOMPLEX 13	I often find it too complex for me to understand and use the TPC-OHCIS application.	2.99 (1.019)
ASEC^e^ 14	I feel secure in using the TPC-OHCIS application, keying in patients’ data, and sharing it across my organization.	2.05 (0.855)
ASEC 15	I would feel safe using the TPC-OHCIS application to retrieve patient data.	2.80 (0.754)
ASEC 16	I am concerned about data patient leakage.	2.07 (0.785)
ASEC 17	I am concerned about how much I can trust the vendor.	1.93 (0.751)
BCHAMP^f^ 18	A specified liaison officer will provide useful information to top managers and vendors about the TPC-OHCIS application faulty.	2.02 (0.736)
BCHAMP 19	A specified liaison officer plays a role in upgrading the TPC-OHCIS application for users’ needs.	2.01 (0.760)
BCHAMP 20	A specified liaison officer has a good relationship with both vendors and top managers.	2.22 (0.851)
BCHAMP 21	A specified liaison officer can bring staff to use the TPC-OHCIS application well.	2.41 (0.972)
BCHAMP 22	A specified liaison officer provides training/courses for the users a few times a year.	2.54 (0.895)
BINFRA^g^ 23	We have enough computers for the TPC-OHCIS application use.	2.27 (0.743)
BINFRA 24	We have a reliable computer network in our use.	2.11 (0.767)
BINFRA 25	Appropriate hardware, software, and network infrastructure were in place before TPC-OHCIS implementation.	1.90 (0.749)
BINFRA 26	We have integrated IS^h^ applications encompassing different functional areas (laboratory and pharmacy).	2.05 (0.775)
BTP^i^ 27	Top management always supports and encourages the use of the TPC-OHCIS application for job-related tasks.	2.15 (0.785)
BTP 28	Top management provides most of the necessary help and resources to enable people to use the TPC-OHCIS system.	2.17 (0.746)
BTP 29	Top management provides good access to hardware when staff need it.	2.15 (0.732)
BTP 30	Top management gives feedback to vendors on every dismayed or unsatisfactory comment from staff.	2.44 (0.822)
BTP 31	Top management provides good access to the TPC-OHCIS application when staff need it.	2.55 (0.842)
BFIN^j^ 32	There is enough financial aid from the organization for the coordination of the system implementation.	2.44 (0.901)
BFIN 33	I find difficulties in using the TPC-OHCIS application because it cannot be upgraded due to not having enough budget.	2.75 (0.814)
BFIN 34	Enough computers are available to access the TPC-OHCIS application.	2.29 (0.743)
BFIN 35	We easily obtain obsolete computer replacements.	2.28 (0.729)
CVEN^k^ 36	Vendors entertain each of our complaints dutifully.	2.41 (0.704)
CVEN 37	The vendor can upgrade the TPC-OHCIS according to our needs.	2.41 (0.771)
CVEN 38	The system vendor attends technical meetings quite frequently.	3.45 (0.819)
CVEN 39	I have a platform to voice out problems regarding the TPC-OHCIS application directly to the vendor.	3.39 (0.805)
DPTC^l^ 40	I do not know how to use computers.	3.19 (0.874)
DPTC 41	I never used to work online.	2.11 (0.849)
DPTC 42	I need people’s help to use a computer.	2.15 (0.859)
DPTC 43	I like to work using the online system.	2.29 (0.836)
DPTC 44	The TPC-OHCIS application is easy to operate.	2.33 (0.794)
DEISK^m^ 45	I have enough training before working with the TPC-OHCIS application.	2.25 (0.836)
DEISK 46	It took me only a few days before I could master the TPC-OHCIS application well.	2.85 (0.920)
DEISK 47	The TPC-OHCIS facilitates task management.	2.63 (0.912)
DEISK 48	The TPC-OHCIS application is hard to use.	2.61 (0.900)
DEISK 49	I have to open many interfaces just to key in 1 patient’s data.	2.05 (0.730)
DEISK 50	The TPC-OHCIS application takes much time because I have to open so many interfaces.	2.50 (0.715)
DCIT^n^ 51	I have confidence in my ability to operate the TPC-OHCIS application.	2.60 (0.725)
DCIT 52	I have the expertise regarding Information technology to provide valuable knowledge to the organization.	2.23 (0.737)
DCIT 53	It does not make any difference whether I add/share knowledge with others related to the use of the TPC-OHCIS application.	2.22 (0.688)
DCIT 54	I feel that other employees can provide more valuable knowledge about the system’s use.	2.22 (0.688)
DCIO^o^ 55	The ITO^p^ is actively considering the introduction of new technology to solve to organization’s problem.	2.34 (0.672)
DCIO 56	The ITO tries to keep a technological leading edge by adopting new technology early.	2.84 (0.823)
DCIO 57	The ITO tends to take risks in the decision-making of new technology introduction.	2.80 (0.847)
DPEU^q^ 58	I often become confused every time I use the TPC-OHCIS application.	2.11 (0.810)
DPEU 59	Interacting with the TPC-OHCIS application is frequently frustrating.	2.18 (0.768)
DPEU 60	I find that the TPC-OHCIS application makes my job easier.	2.28 (0.846)
DPEU 61	The TPC-OHCIS application provides useful guidance in performing tasks.	2.06 (0.764)
DPU^r^ 62	My job would be hard to perform without the TPC-OHCIS application.	2.13 (0.835)
DPU 63	Using the TPC-OHCIS application improves my job performance.	2.18 (0.767)
DPU 64	Using the TPC-OHCIS application saves me time.	2.13 (0.851)
DPU 65	Using the TPC-OHCIS application supports critical aspects of my job (e.g, retrieving patients with missed treatment).	2.17 (0.782)

^a^ARA: domain A, relative advantage.

^b^ACOM: domain A, compatibility.

^c^VEKPRO: vector program for reporting vector-borne diseases and outbreaks.

^d^ACOMPLEX: domain A, complexity.

^e^ASEC: domain A, security.

^f^BICHAMP: domain B, champion.

^g^BINFRA: domain B, infrastructure.

^h^IS: information system.

^i^BTP: domain B, top management support.

^j^BFIN: domain B, financial support.

^k^CVEN: domain C, vendor.

^l^DPTC: domain D, perceived technical competence.

^m^DEISK: domain D, employee information system knowledge.

^n^DCIT: domain D, competency of employee’s IT.

^o^DCIO: domain D, chief information officer innovativeness.

^p^ITO: IT officer.

^q^DPEU: domain D, perceived ease of use.

^r^DPU: domain D, perceived usefulness.

### Statistical Analysis

**Figure 2:**
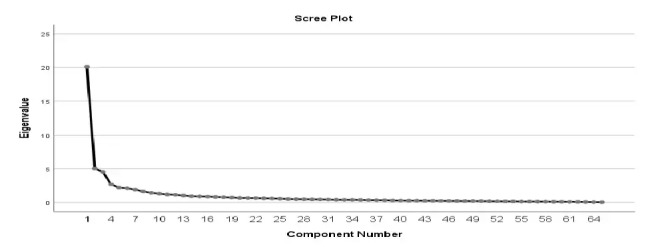
Scree plot revealing the 13-factor components extracted from principal component analysis with varimax rotation.

The 65-item TPC-OHCIS questionnaire was created based on a mix of items adapted from various sources in the development of the questionnaire. It was adapted by a combination of the TOE theory [31], the HOT-fit model [34,35], the DOI theory [36], and the TAM theory [32,33]. The content validity was assessed by experts according to the 4 scales (ie, technology, organization, environment, and human), as highlighted in the previous studies [33-35]. In this study, we explored the constructs generated from this initial group of 65 items, which were developed based on their multidimensional nature. An exploration of the subdimensions based on the 65-item TPC-OHCIS questionnaire at the initial phase found that there were 13 factors, without being restricted to 4 domains. Exploratory factor analysis (EFA) determined the quantity of components (or themes) that emerged for the questionnaire items. The process involved grouping the measurements of comparable themes. The scree plot reveals that there were 13 components in a 65-item questionnaire (Figure 2).

A PCA with varimax rotation yielded 13 components with an eigenvalue of >1. The eigenvalue for the first component was 17.71 and accounted for 27% of the variance. The difference between the first and second components was 11.98, while the subsequent eigenvalues were small (4.91, 4.64, and 3.40). The Kaiser-Meyer-Olkin value was 0.908, which showed sample adequacy. The Bartlett test of sphericity value for the approximate chi-square was *χ*^2^_2080_^=^18,219.9; this was significant (*P*=.001).

Table 4 shows that the eigenvalues from 1.01 to 17.71, corresponding to the extraction sums of squared loadings of 13 subscales of the questionnaire. The highest eigenvalue accounted for 17.71% of the total variance, while the lowest eigenvalue explained 1.01%. The result also shows that the extraction eigenvalue does not vary much from the rotation eigenvalue. Therefore, 65 items loaded strongly on 13 factors at the extraction level.

Table S1 in Multimedia Appendix 4 provides the scale construct and its item. All 13 components had acceptable internal reliability (Cronbach α>0.7). All construct eigenvalues were >1. The deleted items were 14 and 15 of domain A, relative advantage, and 56 and 57 of domain D, chief information officer innovativeness, and the deletions were done because their factor loadings were <0.6. After discussing with the experts, questions for items 56 and 57 of domain D, chief information officer innovativeness, were rephrased for clarity. Question for item 15 of domain A, relative advantage, was omitted, and question for item 14 of the same domain remained as it was.

Table S1 in Multimedia Appendix 4 provides a detailed breakdown of various items and their corresponding domain scores, reflecting their relative contribution to each domain. The items are identified by numbers and grouped under distinct domains. For instance, items 18, 19, 22, 20, 21, 16, 17, and 14 display scores ranging from 0.493 to 0.854, indicating their relevance within a particular domain. Similarly, items 63, 65, 62, 64, 61, 60, 59, and 58 exhibit scores from 0.636 to 0.807 and, therefore, were grouped in another domain. The third domain was categorized based on items 40, 42, 44, 43, 41, 38, and 39, with scores ranging from 0.701 to 0.927. The fourth domain includes items 48, 46, 49, 47, 45, and 50, with scores between 0.0715 and 0.829, and the last domain covers items 27, 31, 30, 29, and 28, with scores ranging from 0.773 to 0.822.

**Table 4:** The total variance explained.

Factor	Initial eigenvalues			Extraction sums of squared loadings		
	Total, n	Variance (%)	Cumulative (%)	Total, n	Variance (%)	Cumulative (%)
1	18	27.2	27.2	18	27.2	27.2
2	6	8.8	36.1	6	8.8	36.1
3	5	7.6	43.6	5	7.6	43.6
4	5	7.1	50.7	5	7.1	50.7
5	3	5.2	55.9	3	5.2	55.9
6	3	4.3	60.3	3	4.3	60.3
7	2	2.9	63.2	2	2.9	63.2
8	2	2.7	65.9	2	2.7	65.9
9	2	2.6	68.6	2	2.6	68.6
10	1	2.2	70.7	1	2.2	70.7
11	1	2.1	72.8	1	2.1	72.8
12	1	1.7	74.5	1	1.7	74.5
13	1	1.6	76.1	1	1.6	76.1

## Discussion

### Principal Findings

This study aimed to validate a specific tool used in health care delivery in Malaysia’s health care system, a new technology telehealth application called TPC-OHCIS, making this evaluation timely. The 65-item TPC-OHCIS questionnaire demonstrated a comprehensive and reliable tool ready to be used for the assessment of the implementation of the digital application system for monitoring the health service program at the PHCs. Monitoring any health care service performance requires a periodic and ongoing operation to ensure that it can be delivered as planned and with good tracking of progress. Evaluation of how the program should be implemented needs to have a valid tool that can give relevant and appropriate results. A validated tool can be used to replicate similar studies in the future for evaluation and comparison.

Developing and validating a locally customized questionnaire for specific program service delivery is important. Therefore, this study used 4 theories, including the HOT-fit model, DOI theory, TOE theory, and TAM theory. The TPC-OHCIS questionnaire was proven valid based on content, face, and construct validities, as well as good psychometric analysis reliability. A total of 4 scales created using PCA did not align with a local study [17] that assessed the hospital information system implementation in Malaysian hospitals. Measuring implementation of technology in different service set-ups and levels of care, at either hospitals or PHCs, should use a customized tool. No similar research paper is available to check for disagreement or benchmarks on other tools, to compare them with the TPC-OHCIS questionnaire validation. Our findings showed that the 13 scales were validated based on the local population working in the PHC with the TPC-OHCIS. In comparison, an earlier study conducted local testing on the hospital information system and identified 4 scales [18]. Our analysis focuses on the MCH services, which are the routine and regularly monitoring health services delivered at PHCs.

A CVI score of 0.90 is generally considered very high and indicative of strong content validity for a measurement tool, particularly in the academic context. Content validity refers to the extent to which the items in a measurement instrument represent the entire range of content that the instrument is intended to measure [39]. A CVI of 0.90 provides strong evidence that the measurement tool has undergone rigorous development and validation processes.

Face validity has been widely used not only in questionnaire development but also in questionnaires that were adopted from other researchers’ tools, which required local forward-backward translation [37-41]. Experts in the field who were recruited to determine face and content validity in our study gave feedback regarding content relevance, wording, quantity of items, the amount of information, and other related issues. The analysis results for the CVI from 10 experts indicated good quality (>0.9), which complies with the recommendations in the literature [41].

Internal reliability assessment using Cronbach α revealed how close the selected items are to one another when measuring the construct [39]. The Cronbach α value of the 65-item TPC-OHCIS questionnaire was 0.90, which exceeded the suggested minimum value of 0.70 [39]. By measuring Cronbach α, the tool’s accuracy and reliability have been verified. Our findings demonstrated that the variables are correlated with the components as constructed through PCA, and it proved that they are internally consistent.

The EFA also determined the TVE for the construct. The TVE illustrated the precision with which the measuring objects and their constituent parts estimate the construct. This study found that the TVE was 76.08%, which exceeded the required minimum of 60%, indicating that the overall variance explained was satisfactory [39]. PCA is the most common EFA method for dimension reduction. The EFA results using varimax rotation with maximum likelihood showed that the TPC-OHCIS questionnaire has 13 subscales.

The study may provide evidence-based recommendations for policy makers and health care stakeholders regarding the implementation and optimization of the TPC-OHCIS in the MCH service delivery monitoring. By identifying key factors influencing the TPC-OHCIS implementation and effectiveness, the study offers actionable insights into policy development, resource allocation, and quality improvement efforts aimed at strengthening MCH care systems. The study may contribute methodological innovations to the field of TPC-OHCIS evaluation, such as novel approaches to questionnaire development, validation, and implementation. By documenting the methodological processes and challenges encountered in assessing the TPC-OHCIS questionnaire validation, the study adds to the methodological toolkit available to researchers and practitioners working in the field of digital health.

Overall, the TPC-OHCIS study offers novel insights into the evaluation of the TPC-OHCIS, which focuses on the MCH services, highlighting the importance of tailored assessment tools, cross-cultural adaptation, policy considerations, and methodological innovations. These insights contribute to a deeper understanding of the complexities and opportunities associated with the integration of digital health technologies into health care delivery and provide a foundation for future research and practice in this area. The TPC-OHCIS questionnaire is validated for evaluating other health information systems, as it takes into account the outcome aspects of health services, technology, organizations, vendors, and human resources. Future researchers can replicate studies to validate the findings of the TPC-OHCIS questionnaire in different populations, settings, and contexts. By replicating studies using a valid and reliable tool with diverse samples, researchers can assess the generalizability and robustness of the questionnaire across various demographic, cultural, and organizational contexts.

### Suggestions to Stakeholders

There is a need to integrate machine learning and artificial intelligence into the TPC-OHCIS so that data can be extracted faster and can be used in line with precision medicine. The validated TPC-OHCIS questionnaire can help stakeholders make effective evidence-based decisions in managing patient-centered care. The system needs to be more stable, easier to use, and faster in terms of data collection, without the need for complicated training, and easy to download from the system itself. Awareness related to the importance of complete and accurate data from an organizational perspective needs to be understood at the level of clinic users and data administrators because they will then enter, process, and ensure that the data entered are complete and accurate. These crucial issues were addressed in our study during the content and face validity assessment of the tool.

The COVID-19 pandemic made us see the need for the TPC-OHCIS to be rolled out to wider PHCs in Malaysia. With <10% of the TPC-OHCIS currently implemented, the vision to having a digital health system in Malaysia by the year 2030 needs to be facilitated. The TPC-OHCIS should be developed as a comprehensive package that includes embedded training and health education to facilitate its delivery. The issue of data security can be compared to the concept of banking system apps in Malaysia, where users only need to download the app on their mobile phones to share information with other health care providers, unlike the TPC-OHCIS, which ensures continuity of care. Otherwise, printing a duplicate report is sufficient for the time being while the country works on transitioning all clinics into a digital system.

### Study Strength

The study demonstrates the development of a tailored questionnaire specifically designed to assess the implementation process and the effectiveness of the TPC-OHCIS in the context of MCH health services at the primary care level. This tailored approach acknowledges the unique challenges and complexities of MCH service delivery and highlights the importance of designing measurement tools that are sensitive to the needs of this population. The study determined comprehensive items to measure the integration implementation of the TPC-OHCIS, which is one of the digital health technologies focusing on priority services, such as MCH service delivery at the primary care level. The study sheds light on the practical considerations, barriers, and facilitators associated with the adoption and use of digital health solutions in MCH care settings. The study offers insights into the cross-cultural adaptability assessment tools, particularly in diverse multiethnicity population settings. By evaluating the linguistic and cultural appropriateness of the TPC-OHCIS questionnaire, the study highlights the importance of considering cultural context and language preferences in developing and validating measurement instruments for MCH services.

### Limitations of This Study

This study was conducted in 2021 when movement control orders were implemented in Malaysia. The study was conducted entirely online, and all surveys were distributed to the respondents’ superiors without proper briefing about the survey and the overall idea of the research except for study information highlighted at the beginning of the online survey questionnaire form. Therefore, each respondent answered as they saw fit, without recognizing the true meaning of the survey. In addition, the deployment of staff during the COVID-19 pandemic could have caused fatigue among them, which was likely to cause them to answer the questions lightly without focus. Meanwhile, the distribution of questionnaires through Google Forms contributed to the risk of bias among respondents when there was no supervision or explanation that could be given as a guide to answering the questionnaire.

### Conclusions

The psychometric validation process of the questionnaire was done comprehensively for all iterative stages, including initial reliability tests, potential modifications based on these tests, and integration with existing digital health assessment frameworks. The novelty of the study lies in its unique approach to conceptualizing the implementation of the TPC-OHCIS. This may offer new insights into the integration of life stage records, with a focus on priority services at PHCs, such as MCH services, for its development. Cross-cultural adaptability was considered to ensure wider applicability, and the rigor of the process was demonstrated in part by high CVI scores, which is important in an academic research setting. Likert scale points were carefully chosen to capture nuanced responses, and while the TPC-OHCIS has certain academic limitations, its robustness paves the way for future research on policy implications and telehealth. Comparison with existing literature establishes its validity and reliability, showing its potential impact on policy development.

## Appendix

Multimedia Appendix 1 Evidence table.

Multimedia Appendix 2 Tele-Primary Care Oral Health Clinical Information System questionnaire.

Multimedia Appendix 3 Expert review analysis.

Multimedia Appendix 4 Domain construct according to item question.

## Abbreviations

CVI: content validity index
DOI: diffusion of innovation
EFA: exploratory factor analysis
HCW: health care worker
HOT-fit: human organization technology-fit
MCH: maternal and child health
PCA: principal component analysis
PHC: primary health care clinic
S-CVI: scale-level content validity index
TAM: technology acceptance model
TOE: technology-organization-environment
TPC-OHCIS: Tele-Primary Care Oral Health Clinical Information System
TVE: total variance explained
