# Supplementary material for: Evaluating the Development, Reliability, and Validation of the Tele-Primary Care Oral Health Clinical Information System Questionnaire: Cross-Sectional Questionnaire Study
Source: JMIR Hum Factors. 2025 Jan 29;12:e53630. doi: 10.2196/53630 (PMC11822314; doi:10.2196/53630)
Supplement: Multimedia Appendix 1 [file humanfactors_v12i1e53630_app1.pdf]

Appendix 1: evidence table

Authors, title of the article	Questionnaire Item, domain, theories	Strength and limitation
Abidi SS, Goh A, Yusoff Z.<br>Title: Telemedicine and medical informatics in the multimedia super corridor: the Malaysian vision	Describe the goal of transforming the Malaysian healthcare delivery system to digital health (telehealth). It listed the components and planning approach toward patient-centered care. It highlights the four domains indirectly related to (1) technology, (2) organization, (3) environment, and (4) human.	Strength:<br>Give insight on the direction, demands, and need to implement telehealth.<br><br>Limitation:<br>No specification on the process of care involved in the implementation of the telehealth application system. It is a review paper.
Mudaris ISM.<br>Title: Digitalised Health Records: Does Malaysia Need It?	Discuss on benefits and implications of digital health records. It has outlined the process of digital health records in ensuring continuity of care and patient advocacy using a few disease examples and comparisons among countries that have implemented it. It highlights the domain (1) technology, (2) organization, (3) environment, and (4) human.	Strength:<br>Give an idea of digital records implementation issues that need to be explored.<br><br>Limitation:<br>This is a review article with no specific methodology design.
Ibrahim AA, Ahmad Zamzuri M'I, Ismail R, Ariffin AH, Ismail A, Muhamad Hasani MH, Abdul Manaf MR.<br>Title: The role of electronic medical records in improving health care quality: A quasi-experimental study.	Describe TPC-OHCIS planning for PHC used. The study presented findings on patient satisfaction using the TPC-OHCIS using a validated questionnaire PSQ-18.	Strength:<br>Assessment of patient satisfaction on TPC-OHCIS. It is a primary research.<br><br>Limitation:<br>Not reporting on healthcare worker satisfaction using the TPC-OHCIS. No specific domains mention TPC-OHCIS implementation.
World Health Organization, Regional Office for the Western Pacific.<br>Title: Malaysia Health System Review	It described healthcare system reform and the transition to digital health. It highlights the importance of domain (1) technology, (2) organization, (3) environment, and (4) human for the national policy of telehealth.	Strength:<br>Provide a national situation view on readiness to transform to digital health.<br><br>Limitation:<br>This is a review document.
Marzuki NM, Ismail S, Abdul Mohsein NAS, Ehsan FZ.	This is a study protocol that assesses the telehealth	Strength:

Title: Evaluation of Telehealth implementation in government primary health clinics-A study protocol.	implementation in the pilot study Putrajaya and Kempas health clinics based on domain: including changes in:<br>1) organisational productivity;<br>2) effectiveness and safety; and<br>3) efficiency indicators.	It focuses on domain interest for the evaluation of telehealth implementation.<br><br>Limitation:<br>The study protocol was published in 2012 whereby many evolutions of the telehealth system are already in the pipeline at present. The study does not focus on TPC-OHCIS implementation. Therefore, the questionnaire used does not outline the current TPC-OHCIS.
Ministry of Health Malaysia. Title: Telemedicine Flagship Application. Malaysia's Telemedicine Blueprint: Leading Healthcare into the Information Age.	A blueprint document on Malaysia's telemedicine plan. It outlines the important domain: humans and technology.	Strength:<br>The goal and objective of telemedicine and planning are clearly stated.<br><br>Limitation:<br>This is a policy document that has no specific description of the process of implementation and its strategies.
Mohan J, Raja Yaacob RR. Title: The Malaysian Telehealth Flagship Application: a national approach to health data protection and utilisation and consumer rights	Description of the telehealth application components and its planning process for implementation. It elaborates the domains interest (1) technology, (2) organization, (3) environment, and (4) human.	Strength:<br>A policy document for reference of telehealth implementation goals.<br><br>Limitation:<br>A review document
Maon SN, Edirippulige S. Title: An overview of the national telehealth initiative in Malaysia.	This document described the telehealth initiative and domains of interest (1) technology, (2) organization, (3) environment, and (4) human.	Strength:<br>The initiatives are well described with supporting evidence.<br><br>Limitation:<br>A review documents
Family Health Development Division.	This website has an overview of the TPC-OHCIS and its use in family health services.	Strength:<br>Elaborate on TPC-OHCIS strategies for the implementation in brief.

Title: Teleprimary Care – Oral Health Clinical Information System (TPC-OHCIS).		Limitation:<br>No update on the recent progress of its implementation at the PHCs.
MIMOS.<br>Title: TPC-OHCIS–Teleprimary Care and Oral Health Clinical Information System	This website belongs to the provider of the TPC-OHCIS application. It describes the features of the application.	Strength:<br>Features of TPC-OHCIS described.<br><br>Limitation:<br>Not updated on its present implementation.
Marzuki NM, Ismail S, Abdul Mohsein NAS, Ehsan FZ, Chan CK, Ng CW.<br>Title: Integrating Information and Communication Technology for Health Information System Strengthening: A Policy Analysis.	A situational analysis of the implementation of the telehealth system in Malaysia health facilities. It discussed on domain of interest (1) technology, (2) organization, (3) environment, and (4) human.	Strength:<br>A policy analysis article that described factors affecting the telehealth implementation process.<br><br>Limitation:<br>No questionnaire item was used. This is a qualitative research and data obtained from key informants' interviews as case studies.
Khobi JAM, Mtebe JS, Mbelwa JT.<br>Title: Factors Influencing District Health Information System Usage in Sierra Leone: A Study Using the technology-organization-environment framework	Study findings: top management support, perceived benefits, security and privacy, and compatibility are strongly associated with system usage. Challenges identified Internet connectivity, data protection security policies and guidelines, and competent manpower shortage. It described the components and items of the domain of interest: 1) technology, (2) organization, (3) environment, and (4) human. The questionnaire validation process is described.	Strength:<br>It used the Technology-Organization-Environment theory in its methodological framework. It addressed the challenges faced using the DHIS2 besides infrastructural and system factors. A primary research using a validated questionnaire.<br><br>Limitation:<br>The study was conducted not in Malaysia and focused on district health information systems.
Alsharo M, Alnsour Y, Alabdallah M.<br>Title:How habit affects continuous use: evidence from Jordan's national health information system	It evaluates human behaviors on the health information system usage. The theory employed is based on the technology acceptance model. Specific domains on humans used: perceived ease of use, attitude, intention to continue use, habit	Strength:<br>A primary research focussed on health information usage among the healthcare staff. Used a validated questionnaire.<br><br>Limitation:

	and perceived usefulness HIS were assessed.	Not focussed on the 1) technology, (2) organization, (3) environment domains
Salleh MIM, Abdullah R. & Zakaria N.<br>Title: Evaluating the effects of electronic health records system adoption on the performance of Malaysian health care providers	It evaluated the health information system and electronic medical records implemented in hospitals in Malaysia. Used the DeLone and McLean (D&M) models and technology and technology acceptance models to create a questionnaire for assessing the system usage among healthcare workers.	Strength:<br>A primary research using a validated questionnaire was conducted in Malaysia to assess the healthcare workers' usage.<br><br>Limitation:<br>Evaluate the system used among workers referral hospitals implemented the system.
Ahmadi H, Nilashi M, Ibrahim O.<br>Title: Organizational decision to adopt hospital information system: an empirical investigation in the case of Malaysian public hospitals	Employed the Technology-Organization-Environment (TOE) framework and Human-Organization-Technology (HOT) fit model in the evaluation of the hospital's decision to adopt a Hospital Information System (HIS).	Strength:<br>Primary research conducted in Malaysia uses multiple theories in constructing their questionnaire for evaluation. The questionnaire was validated and presented. The questionnaire was constructed based on domain of interest: 1) technology, (2) organization, (3) environment, and (4) human.<br><br>Limitation:<br>Focussed on a hospital setting.
Zhang X, Yu P, Yan J. et al.<br>Using diffusion of innovation theory to understand the factors impacting patient acceptance and use of consumer e-health innovations: a case study in a primary care clinic.	It evaluated the factors in implementing eHealth. It specifically used the diffusion of innovation models to identify factors of eHealth adoption at the primary healthcare clinics: way of communication on its intervention, the feature used in the innovation, socio-demographic of the user, and awareness of the innovation	Strength:<br>Primary research was conducted at primary healthcare clinics for the adoption of the eHealth implementation.<br><br>Limitation:<br>This is a qualitative study and there is no specific quantitative study tool (questionnaire).
