# Supplementary material for: Evaluating the Development, Reliability, and Validation of the Tele-Primary Care Oral Health Clinical Information System Questionnaire: Cross-Sectional Questionnaire Study
Source: JMIR Hum Factors. 2025 Jan 29;12:e53630. doi: 10.2196/53630 (PMC11822314; doi:10.2196/53630)
Supplement: Multimedia Appendix 2 [file humanfactors_v12i1e53630_app2.docx]

**Teleprimary Care Oral Health Clinical Information System (TPCOHCIS)**

**Borang Soal Selidik Sistem Jagaan Tele Primer, Informasi Klinikal Kesihatan Dental (TPCOHCIS)**

*Instruction: Please mark (√) in the appropriate Likert scale box.*

*Scale 1= Disagree the most, Scale 2= Disagree, Scale 3= Agree, Scale= 4 Agree the most*

Arahan : Sila tandakan (√) pada kotak skala Likert yang berkenaan.

Skala 1= Paling tidak setuju, Skala 2= Tidak Setuju, Skala 3= Setuju, Skala= 4 Paling setuju

| Item | | Likert Scale | | | |
| --- | --- | --- | --- | --- | --- |
|  |  | 1 | 2 | 3 | 4 |
| **A.Technology Domain** | |  |  |  |  |
| ARA | Relative advantage  *Kelebihan relatif* |  |  |  |  |
| 1 | Using the TPCOHCIS system enables me to do my work quickly.  *Kerja saya lebih cepat selesai dengan menggunakan sistem TPCOHCIS* |  |  |  |  |
| 2 | Using the TPCOHCIS system improves my quality of work.  *Kerja saya lebih berkualiti dengan menggunakan sistem TPCOHCIS.* |  |  |  |  |
| 3 | Using the TPCOHCIS system enhances my effectiveness on the job.  *Sistem TPCOHCIS meningkatkan keberkesanan kerja saya.* |  |  |  |  |
| 4. | Using the TPCOHCIS system increases my productivity.  *Sistem TPHOHCIS meningkatkan produktiviti saya.* |  |  |  |  |
| 5. | Using the TPCOHCIS system makes my job easier.  *Kerja saya lebih senang menggunakan sistem TPCOHCIS.* |  |  |  |  |
| ACOM | Compatibility  *Keserasian* |  |  |  |  |
| 6. | TPCOHCIS system application can be easily accessed across multiple platforms (laboratory results, X-Ray, and other related patient data).  *Aplikasi Sistem TPCOHCIS adalah lebih mudah untuk akses kepada pelbagai platform (keputusan makmal, X-Ray dan lain-lain data pesakit)* |  |  |  |  |
| 7. | TPCOHCIS system user interfaces provide transparent access to all platforms *(e-notification, VEKPRO)*  *Halaman depan Sistem TPCOHCIS menyediakan akses untuk memasukkan data terus ke pelbagai platforms (e-notifikasi, VEKPRO).* |  |  |  |  |
| 8. | Data received from other devices (tablet /laptop/smartphone) outside health facilities in the TPCOHCIS system application can be easily merged into the database for analysis.  *Data yang dimasukkan melalui telefon/laptop ke dalam sistem applikasi TPCOHCIS boleh disatukan dalam database untuk analisa* |  |  |  |  |
| 9. | Information is shared seamlessly across our organization regardless of the location.  *Data di kongsi dan dapat di akses oleh anggota terlibat denganTPCOHCIS walaupun berada di lapangan.* |  |  |  |  |
| ACOMPLEX | Complexity  *Kerumitan* |  |  |  |  |
| 10. | I do not know enough about the TPCOHCIS system to handle my job satisfactorily.  *Saya susah nak faham penggunaan Sistem TPCOHCIS untuk menjalankan kerja dengan baik.* |  |  |  |  |
| 11. | I need a long time to understand and get familiar to use the TPCOHCIS system.  *Saya ambil masa yang lama untuk pandai menggunakan Sistem TPCOHCIS.* |  |  |  |  |
| 12. | I do not find enough time to study and upgrade my technology skills before using the TPCOHCIS system.  *Saya tak ada masa yang cukup untuk belajar dan mahir sebelum menggunakan sistem TPCOHCIS.* |  |  |  |  |
| 13. | I often find it too complex for me to understand and use this TPCOHCIS system.  *Saya merasakan Sistem TPCOHCIS terlalu rumit dan kompleks.* |  |  |  |  |
| ASEC | Security concern  *Isu Keselamatan* |  |  |  |  |
| 14. | I feel secure in using the TPCOHCIS system, keying in patients’ data, and sharing it across my organization.  *Saya yakin keselamatan data pesakit yang dimasukkan dalam Sistem TPCOHCIS dan dikongsi dalam organisasi saya.* |  |  |  |  |
| 15. | I would feel totally safe using the TPCOHCIS system to retrieve patients’ data.  *Saya rasa data pesakit selamat untuk di muat turun dari Sistem TPCOHCIS.* |  |  |  |  |
| 16. | I am concerned about data patient leakage.  *Saya prihatin kejadian kecurian data pesakit.* |  |  |  |  |
| 17. | I am concerned about how much I can trust the vendor.  *Saya berhati hati dalam memberi sepenuh kepercayaan kepada vendor berkaitan data pesakit.* |  |  |  |  |
| **B.Organization Domain** | |  |  |  |  |
| BCHAMP | Presence of specified liaison officer  Pegawai penghubung rujukan. |  |  |  |  |
| 18. | A specified liaison officer will provide useful information to top managers and vendors about the TPCOHCIS system faulty.  *Pegawai penghubung rujukan akan memberikan maklumbalas kepada Pembekal dan pengurusan tertinggi berkaitan masalah Sistem TPCOHCIS.* |  |  |  |  |
| 19. | A specified liaison officer plays a role in upgrading the TPCOHCIS system for users’ needs.  *Pegawai penghubung rujukan memainkan peranan yang penting bagi menambah fungsi Sistem TPCOHCIS.* |  |  |  |  |
| 20. | A specified liaison officer has a good relationship with both vendors and top managers.  *Pegawai penghubung rujukan mempunyai hubungan yang baik dengan pembekal dan pengurusan tertinggi.* |  |  |  |  |
| 21. | A specified liaison officer is able to bring staff to use the TPCOHCIS system well.  *Pegawai penghubung rujukan berkebolehan untuk mengajak staf menggunakan Sistem TPCOHCIS.* |  |  |  |  |
| 22. | A specified liaison officer provides training/courses for the users a few times a year.  *Pegawai penghubung rujukan menyediakan latihan /kursus berkaitan sistem beberapa kali setahun.* |  |  |  |  |
| BINFRA | Infrastructure  *Infrastruktur* |  |  |  |  |
| 23. | We have enough computers for the TPCOHCIS system use.  *Kami mempunyai komputer yang mencukupi untuk menggunakan Sistem TPCOHCIS.* |  |  |  |  |
| 24. | We have a reliable computer network in our use.  *Kami mempunyai jaringan rangkaian komputer yang stabil.* |  |  |  |  |
| 25. | Appropriate hardware, software, and network infrastructures were in place prior to the TPCOHCIS system implementation.  *Semua keperluan komputer, perisian sistem, rangkaian jaringan telah lengkap sebelum diimplementasikan.* |  |  |  |  |
| 26 | Presence of integrated Information System applications encompassing different functional areas (retrieve Lab results, X-Ray, Pharmacy).  *Terdapat sistem applikasi integrasi informasi merangkumi pelbagai keperluan fungsian (contoh mendapatkan keputusan makmal, X-Ray, Farmasi).* |  |  |  |  |
| BTP | Top Management Support  *Sokongan pegawai atasan*. |  |  |  |  |
| 27. | Top Management always supports and encourages the use of the TPCOHCIS system for job-related tasks.  *Pegawai atasan sentiasa menyokong penggunaan sistem TPCOHCIS untuk aktiviti kerja berkaitan.* |  |  |  |  |
| 28. | Top management provides most of the necessary help and resources to enable staff to use the TPCOHCIS system.  *Pegawai atasan menyediakan keperluan dan sumber bagi pekerjanya menggunakan Sistem TPCOHCIS.* |  |  |  |  |
| 29. | Top management provides good access to hardware when staffs need them.  *Pegawai atasan memberikan akses yang baik kepada pekerjanya yang memerlukan perkakasan komputer berkaitan.* |  |  |  |  |
| 30. | Top management gives feedback to vendors on every dismay or unsatisfactory comment from staff  *Pegawai atasan memberikan maklumbalas dari stafnya berkaitan masalah sistem kepada pembekal.* |  |  |  |  |
| 31. | Top management provides good access to the TPCOHCIS system when staff needs it.  *Pegawai atasan menyediakan kemudahan internet yang baik kepada stafnya semasa menggunakan sistem.* |  |  |  |  |
| BFIN | Financial resources  *Sumber kewangan* |  |  |  |  |
| 32. | There is enough financial aid from the organization for the coordination of the system implementation.  *Organisasi menyalurkan peruntukan yang mencukupi untuk tujuan menyelaras implementasi sistem.* |  |  |  |  |
| 33. | I find difficulties to use the TPCOHCIS system because it cannot be upgraded due to not having enough budget.  *Saya rasa susah menggunakan sistem TPCOHCIS kerana sistem ini tidak dapat dinaiktaraf atas masalah peruntukan kewangan.* |  |  |  |  |
| 34. | Enough computers are available to access the TPCOHCIS system.  *Komputer mencukupi untuk menggunakan Sistem TPCOHCIS.* |  |  |  |  |
| 35. | We easily get obsolete computer replacements.  *Kami mudah mendapatkan penggantian komputer yang telah rosak.* |  |  |  |  |
| **Environment Domain** | |  |  |  |  |
| CVEN | Vendor support  *Sokongan dari pembekal* |  |  |  |  |
| 36. | The vendor entertains each one of our complaints dutifully.  *Pembekal menangani setiap aduan dengan bertanggungjawab.* |  |  |  |  |
| 37. | The vendor is able to upgrade the TPCOHCIS system according to our needs.  *Pembekal boleh menaiktaraf sistem TPCOHCIS mengikut kesesuaian/keperluan kerja kami.* |  |  |  |  |
| 38. | The system vendor attended the technical meetings quite frequently.  *Pembekal kerap masuk mesyuarat teknikal bersama..* |  |  |  |  |
| 39. | I have a platform to voice out problems regarding the TPCOHCIS system direct to the vendors.  *Saya mempunyai platform untuk mengutarakan keluhan berkaitan sistem TPCOHCIS terus kepada pembekal.* |  |  |  |  |
| **Human Domain** | |  |  |  |  |
| DPT | Staff competency on Information technology System  *Kompetensi staf terhadap sistem informasi teknologi*. |  |  |  |  |
| 40. | I don’t know how to use a computer.  *Saya tak pandai guna komputer.* |  |  |  |  |
| 41. | I never use to work online.  *Saya tidak pernah melakukan tugasan secara atas talian.* |  |  |  |  |
| 42. | I need people’s help to use a computer.  *Saya perlu bantuan orang lain untuk menggunakan komputer.* |  |  |  |  |
| 43. | I like to work using the online system  *Saya suka kerja mengunakan sistem atas talian.* |  |  |  |  |
| 44. | The TPCOHCIS system is easy to be operated.  *Operasi Sistem TPCOHCIS mudah dikendalikan.* |  |  |  |  |
| DEIS | Knowledge on the TPCOHCIS system  *Pengetahuan mengenai sistem TPCOHCIS* |  |  |  |  |
| 45. | I have enough training/courses before working with the TPCOHCIS system.  *Latihan/kursus yang diberikan mencukupi sebelum saya mula menggunakan sistem TPCOHCIS.* |  |  |  |  |
| 46. | It took me only a few days before I can master the TPCOHCIS system well.  *Saya hanya mengambil masa beberapa hari sahaja untuk mahir mengendalikan sistem TPCOHCIS.* |  |  |  |  |
| 47. | The TPCOHCIS system facilitates task management.  *Sistem TPCOHCIS memudahkan pengurusan kerja.* |  |  |  |  |
| 48. | The TPCOHCIS system is hard to use.  *Sistem TPCOHCIS susah dikendalikan* |  |  |  |  |
| 49 | I have to open many interfaces just to key in one patient’s data.  *Saya terpaksa membuka banyak interface/ muka untuk menyelesaikan satu kes pesakit.* |  |  |  |  |
| 50 | TPCOHCIS system taking much time because I have to open so many interfaces.  *Sistem TPCOHCIS mengambil masa lama sebab terpaksa membuka banyak interface/muka.* |  |  |  |  |
| DCIT | Clinical information technology competency  *Kepakaran dalam informasi teknologi klinikal* |  |  |  |  |
| 51 | I have confidence in my ability to operate the TPCOHCIS system.  *Saya yakin dengan kemampuan saya mengendalikan Sistem TPCOHCIS.* |  |  |  |  |
| 52 | I have the expertise regarding Information technology to provide valuable knowledge to the organization  Saya mempunyai kemahiran berkaitan teknologi maklumat yang mungkin berguna kepada organisasi. |  |  |  |  |
| 53 | It doesn’t make any difference whether I add/ share knowledge with others related to the usage of the TPCOHCIS Systems.  *Tiada perbezaanpun akan berlaku samada saya berkongsi/ menambah pemahaman berkaitan penggunaan sistem TPCOHCIS* |  |  |  |  |
| 54 | I feel that other employees can provide more valuable knowledge about the system’s use.  *Orang lain boleh memberikan pendidikan yang lebih berguna berkaitan penggunaan sistem.* |  |  |  |  |
| DCIO | Perceived Innovativeness of Information Technology Officer (ITO)  *Persepsi Inovatif Pegawai Teknologi Maklumat (PTM*). |  |  |  |  |
| 55 | The ITO is actively considering the introduction of new technology to solve to organization’s problem.  *PTM sentiasa memperkenalkan fungsi baru bagi membantu menyelesaikan masalah yang dihadapi oleh organisasi.* |  |  |  |  |
| 56 | The ITO tries to keep a technological leading edge by adopting new technology early.  PTM sentiasa memperkenalkan sistem baru supaya sentiasa menggunakan teknologi terkini. |  |  |  |  |
| 57 | The ITO tends to take risks in the decision-making of new technology introduction.  *PTM cenderung mengambil risiko dalam membuat keputusan memperkenalkan teknologi baru.* |  |  |  |  |
| DPEU | Perceived Ease Use  *Persepsi penggunaan mudah* |  |  |  |  |
| 58 | I often become confused every time I use TPCOHCIS.  *Saya seringkali keliru bila menggunakan sistem TPCOHCIS.* |  |  |  |  |
| 59 | Interacting with the TPCOHCIS system is frequently frustrating.  *Mengendalikan Sistem TPCOHCIS seringkali membuatkan saya kecewa.* |  |  |  |  |
| 60 | I find the TPCOHCIS system makes my job easier.  *Sistem TPCOHCIS memudahkan kerja saya.* |  |  |  |  |
| 61 | The TPCOHCIS system provides useful guidance in performing tasks.  *Sistem TPCOHCIS memberikan bimbingan berguna dalam menjalankan tugasan.* |  |  |  |  |
| DPU | Perceived Usefulness  *Persepsi Kebergunaan* |  |  |  |  |
| 62 | My job would be hard to perform without the TPCOHCIS system.  *Kerja saya lebih susah kalau tak ada Sistem TPCOHCIS.* |  |  |  |  |
| 63 | Using the TPCOHCIS system improves my job performance  *Dengan menggunakan sistem TPCOHCIS meningkatkan prestasi kerja saya.* |  |  |  |  |
| 64 | Using the TPCOHCIS system saves me job time.  *Dengan menggunakan Sistem TPCOHCIS, saya dapat menjimatkan masa kerja.* |  |  |  |  |
| 65 | Using the TPCOHCIS system supports critical aspects of my job (e.g. retrieving patients with missed treatment).  *Sistem sokongan TPCOHCIS menyelesaikan kerja-kerja sukar saya (contoh mencari pesakit yang cicir rawatan).* |  |  |  |  |

Note: There are 15 items (10,11,12,13,33,40,41,42,48,49,50,53,54,58 and 39) that are negative items. The Likert score obtained should be reversed for scoring analysis.

Suggestion for analysis:

There were 65 items-questionnaire with four Likert scale (1=highly disagree, 2=disagree, 3=agree, 4=highly agree). To calculate the mean total sum score of each 4 domains (**Technology, Organization, Environmental, Human)** and the mean total score for each of 13 subdomains:

Technology domain

- Relative advantage (5 items)
- Compatibility(4 items)
- Complexity(4 items)
- Security concern (4 items)

Organization domain

- Presence of specified liaison officer (5 items)
- Infrastructure(4 items)
- Top Management Support(5 items)
- Financial resources(4 items)

Environmental domain:

- Vendor support (4 items)

Human Domain

- Staff competency on Information technology System (12 items)
- Knowledge on the TPCOHCIS system (6 items)
- Clinical information technology competency(4 items)
- Perceived Innovative Information Technology Officer (ITO)(3 items)
- Perceived Ease Use (4 items)
- Perceived Usefulness (4 items)
