# Supplementary material for: Evaluating the Development, Reliability, and Validation of the Tele-Primary Care Oral Health Clinical Information System Questionnaire: Cross-Sectional Questionnaire Study
Source: JMIR Hum Factors. 2025 Jan 29;12:e53630. doi: 10.2196/53630 (PMC11822314; doi:10.2196/53630)
Supplement: Multimedia Appendix 4 [file humanfactors_v12i1e53630_app4.docx]

Multimedia Appendix 4: Principal component analysis of TPC-OHCIS Questionnaire items

| Item number | Domain 1  (eigenvalues) | Domain 2  (eigenvalues) | Domain 3  (eigenvalues) | Domain 4  (eigenvalues) | Domain 5  (eigenvalues) | Domain 6  (eigenvalues) | Domain 7  (eigenvalues) | Domain 8  (eigenvalues) | Domain 9  (eigenvalues) | Domain 10  (eigenvalues) | Domain 11  (eigenvalues) | Domain 12  (eigenvalues) | Domain 13  (eigenvalues) |
| --- | --- | --- | --- | --- | --- | --- | --- | --- | --- | --- | --- | --- | --- |
| 18 | .854 |  |  |  |  |  |  |  |  |  |  |  |  |
| 19 | .826 |  |  |  |  |  |  |  |  |  |  |  |  |
| 22 | .811 |  |  |  |  |  |  |  |  |  |  |  |  |
| 20 | .809 |  |  |  |  |  |  |  |  |  |  |  |  |
| 21 | .760 |  |  |  |  |  |  |  |  |  |  |  |  |
| 16 | .741 |  |  |  |  |  |  |  |  |  |  |  |  |
| 17 | .706 |  |  |  |  |  |  |  |  |  |  |  |  |
| 14 | .493 |  |  |  |  |  |  |  |  |  |  |  |  |
| 63 |  | .807 |  |  |  |  |  |  |  |  |  |  |  |
| 65 |  | .786 |  |  |  |  |  |  |  |  |  |  |  |
| 62 |  | .773 |  |  |  |  |  |  |  |  |  |  |  |
| 64 |  | .769 |  |  |  |  |  |  |  |  |  |  |  |
| 61 |  | .731 |  |  |  |  |  |  |  |  |  |  |  |
| 60 |  | .702 |  |  |  |  |  |  |  |  |  |  |  |
| 59 |  | .656 |  |  |  |  |  |  |  |  |  |  |  |
| 58 |  | .636 |  |  |  |  |  |  |  |  |  |  |  |
| 40 |  |  | .927 |  |  |  |  |  |  |  |  |  |  |
| 42 |  |  | .859 |  |  |  |  |  |  |  |  |  |  |
| 44 |  |  | .848 |  |  |  |  |  |  |  |  |  |  |
| 43 |  |  | .824 |  |  |  |  |  |  |  |  |  |  |
| 41 |  |  | .799 |  |  |  |  |  |  |  |  |  |  |
| 38 |  |  | .766 |  |  |  |  |  |  |  |  |  |  |
| 39 |  |  | .701 |  |  |  |  |  |  |  |  |  |  |
| 48 |  |  |  | .829 |  |  |  |  |  |  |  |  |  |
| 46 |  |  |  | .760 |  |  |  |  |  |  |  |  |  |
| 49 |  |  |  | .745 |  |  |  |  |  |  |  |  |  |
| 47 |  |  |  | .726 |  |  |  |  |  |  |  |  |  |
| 45 |  |  |  | .724 |  |  |  |  |  |  |  |  |  |
| 50 |  |  |  | .715 |  |  |  |  |  |  |  |  |  |
| 27 |  |  |  |  | .822 |  |  |  |  |  |  |  |  |
| 31 |  |  |  |  | .798 |  |  |  |  |  |  |  |  |
| 30 |  |  |  |  | .797 |  |  |  |  |  |  |  |  |
| 29 |  |  |  |  | .774 |  |  |  |  |  |  |  |  |
| 28 |  |  |  |  | .773 |  |  |  |  |  |  |  |  |
| 5 |  |  |  |  |  | .795 |  |  |  |  |  |  |  |
| 3 |  |  |  |  |  | .780 |  |  |  |  |  |  |  |
| 2 |  |  |  |  |  | .773 |  |  |  |  |  |  |  |
| 4 |  |  |  |  |  | .760 |  |  |  |  |  |  |  |
| 1 |  |  |  |  |  | .710 |  |  |  |  |  |  |  |
| 35 |  |  |  |  |  |  | .910 |  |  |  |  |  |  |
| 33 |  |  |  |  |  |  | .878 |  |  |  |  |  |  |
| 34 |  |  |  |  |  |  | .867 |  |  |  |  |  |  |
| 32 |  |  |  |  |  |  | .857 |  |  |  |  |  |  |
| 6 |  |  |  |  |  |  |  | .854 |  |  |  |  |  |
| 9 |  |  |  |  |  |  |  | .801 |  |  |  |  |  |
| 8 |  |  |  |  |  |  |  | .783 |  |  |  |  |  |
| 7 |  |  |  |  |  |  |  | .768 |  |  |  |  |  |
| 12 |  |  |  |  |  |  |  |  | .848 |  |  |  |  |
| 10 |  |  |  |  |  |  |  |  | .836 |  |  |  |  |
| 13 |  |  |  |  |  |  |  |  | .831 |  |  |  |  |
| 11 |  |  |  |  |  |  |  |  | .798 |  |  |  |  |
| 15 |  |  |  |  |  |  |  |  | 258 |  |  |  |  |
| 54 |  |  |  |  |  |  |  |  |  | .935 |  |  |  |
| 51 |  |  |  |  |  |  |  |  |  | .911 |  |  |  |
| 52 |  |  |  |  |  |  |  |  |  | .898 |  |  |  |
| 53 |  |  |  |  |  |  |  |  |  | .873 |  |  |  |
| 24 |  |  |  |  |  |  |  |  |  |  | .810 |  |  |
| 26 |  |  |  |  |  |  |  |  |  |  | .793 |  |  |
| 25 |  |  |  |  |  |  |  |  |  |  | .781 |  |  |
| 23 |  |  |  |  |  |  |  |  |  |  | .774 |  |  |
| 37 |  |  |  |  |  |  |  |  |  |  |  | .755 |  |
| 36 |  |  |  |  |  |  |  |  |  |  |  | .721 |  |
| 55 |  |  |  |  |  |  |  |  |  |  |  |  | .605 |
| 56 |  |  |  |  |  |  |  |  |  |  |  |  | .492 |
| 57 |  |  |  |  |  |  |  |  |  |  |  |  | .430 |

Footnote: Only strong eigenvalues were presented in the cell for variables (item number) that strongly affected and were the contributors to the domain (principle component). All values were strong positive eigenvalues, showing a positive correlation with the variable. The values were present were <1.
